# Medical Insurance Information Systems in China: Mixed Methods Study

**DOI:** 10.2196/18780

**Published:** 2020-09-01

**Authors:** Yazi Li, Chunji Lu, Yang Liu

**Affiliations:** 1 The Institute of Medical Information Chinese Academy of Medical Sciences Beijing China

**Keywords:** medical insurance, medical insurance information system, health information exchange, information infrastructure, big data, policy review, privacy protection

## Abstract

**Background:**

Since the People’s Republic of China (PRC), or China, established the basic medical insurance system (MIS) in 1998, the medical insurance information systems (MIISs) in China have effectively supported the operation of the MIS through several phases of development; the phases included a stand-alone version, the internet, and big data. In 2018, China’s national medical security systems were integrated, while MIISs were facing reconstruction. We summarized China’s experience in medical insurance informatization over the past 20 years, aiming to provide a reference for the building of a new basic MIS for China and for developing countries.

**Objective:**

This paper aims to sort out medical insurance informatization policies throughout the years, use questionnaires to determine the status quo of provincial MIIS-building in China and the relevant policies, provide references and suggestions for the top-level design and implementation of the information systems in the transitional period of China’s MIS reform, and provide a reference for the building of MIISs in developing countries.

**Methods:**

We conducted policy analysis by collecting the laws, regulations, and policy documents—issued from 1998 to 2020—on China's medical insurance and its informatization; we also analyzed the US Health Insurance Portability and Accountability Act and other relevant policies. We conducted a questionnaire survey by sending out questionnaires to 31 Chinese, provincial, medical security bureaus to collect information about network links, system functions, data exchange, standards and specifications, and building modes, among other items. We conducted a literature review by searching for documents about relevant laws and policies, building methods, application results, and other documents related to MIISs; we conducted searches using PubMed, Elsevier, China National Knowledge Infrastructure, and other major literature databases. We conducted telephone interviews to verify the results of questionnaires and to understand the focus issues concerning the building of China’s national MIISs during the period of integration and transition of China's MIS.

**Results:**

In 74% (23/31) of the regions in China, MIISs were networked through dedicated fiber optic lines. In 65% (20/31) of the regions in China, MIISs supported identity recognition based on both ID cards and social security cards. In 55% (17/31) of the regions in China, MIISs at provincial and municipal levels were networked and have gathered basic medical insurance data, whereas MIISs were connected to health insurance companies in 35% (11/31) of the regions in China. China’s MIISs are comprised of 11 basic functional modules, among which the modules of business operation, transregional referral, reimbursement, and monitoring systems are widely applied. MIISs in 83% (20/24) of Chinese provinces have stored data on coverage, payment, and settlement compensation of medical insurance. However, in terms of data security and privacy protection, pertinent policies are absent and data utilization is not in-depth enough. Respondents to telephone interviews universally reflected on the following issues and suggestions: in the period of integration and transition of MISs, close attention should be paid to the top-level design, and repeated investment should be avoided for the building of MIISs; MIISs should be adapted to the health care reform, and efforts should be made to strengthen the informatization support for the reform of payment methods; and MIISs should be adapted for the widespread application of mobile phones and should provide insured persons with more self-service functions.

**Conclusions:**

In the future, the building of China’s basic MIISs should be deployed at the national, provincial, prefectural, and municipal levels on a unified basis. Efforts should be made to strengthen the development of standard codes, data exchange, and data utilization. Work should be done to formulate the rules and regulations for security and privacy protection and to balance the right to be informed with the mining and utilization of big data. Efforts should be made to intensify the interconnectivity between MISs and other health systems and to strengthen the application of medical insurance information in public health monitoring and early warning systems; this would ultimately improve the degree of trust from stakeholders, including individuals, medical service providers, and public health institutions, in the basic MIISs.

## Introduction

### Background

China’s medical insurance information systems (MIISs) are connected to over 700,000 medical institutions at various levels; cover more than 1.35 billion people [1]; record medical insurance data regarding coverage, payment, claim, and compensation; offer information management tools for the world's largest medical security network; and provide innovation means for health care reform and change in medical insurance systems (MISs). Many countries in the world have built their own national-level medical security information networks. For example, France built the National Health Insurance Inter-regime Information System [2]; the United States established a medical security network that covers all medical service providers nationwide through the Health Information Technology for Economic and Clinical Health Act of 2009 [3]; and South Korea built the National Health Information Database [4], which provides support for the operation of its MIS.

Over the past 20 years, China’s MISs consisted of the following three main parts: (1) medical insurance for urban employees established in 1998, (2) the New Rural Cooperative Medical System (NRCMS) for rural residents established in 2003, and (3) medical insurance for urban residents established in 2008. Medical insurance for urban employees and urban residents was overseen by the Ministry of Human Resources and Social Security of the People's Republic of China (PRC), and the NRCMS for rural residents was overseen by the Ministry of Health of the PRC [5]. In 2018, after the completion of China’s system reform and national institutional reform, China’s basic MISs were integrated, and the National Healthcare Security Administration (NHSA) of the PRC was established, while two systems were retained; these two systems were the medical insurance for urban employees and the basic medical insurance for urban and rural residents, resulting from the consolidation of medical insurance for urban residents and the NRCMS. The function of assistance for the disadvantaged was transferred from the Ministry of Civil Affairs of the PRC to the NHSA, the functions of pricing of medical service items and bidding procurement of medicines (ie, drugs) were transferred from the National Development and Reform Commission (NDRC) of the PRC to the NHSA, and the function of collection and payment of medical insurance funds would be implemented by the taxation departments [6,7].

**Figure 1:**
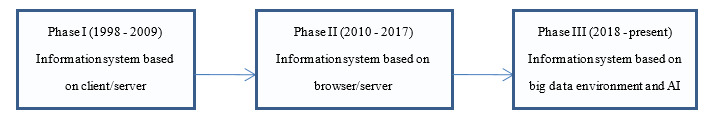
The three phases of the development of China’s medical insurance information systems (MIISs).

Since 1998, the building of China’s MIISs has gone through three phases: Phase I, where the stand-alone version realized the handling of medical insurance business; Phase II, where extensive interconnection was based on the internet; and Phase III, where comprehensive decision-making functions based on big data were realized. China’s MIISs were established on the basis of the pooling level and have the functions of fundraising, payment, online reimbursement and settlement, and capital settlement, among others. Figure 1 shows the three phases of the development of China’s MIISs.

Phase I took place from 1998 to 2009, when local MIISs were established separately in different pooling-based regions in China according to the needs for business development. Over 2000 NRCMS information systems were established in China at the county level, and more than 300 information systems were established at the prefectural or municipal level in China for urban employees and residents. In terms of functions, these information systems mainly functioned to manage the accounts and insurance participation registration for the insured entities, families, and individuals, and they managed fund collection and expenditure. Before 2009, China’s MIISs were based on a stand-alone version [8], which lacked overall planning and design; there existed such serious problems as repeated investment and isolated islands of information systems, while circumstances of poor system interaction and repeated insurance participation occurred from time to time [9].

Phase II took place from 2010 to 2017, when the medical insurance management system tended to be integrated, and MIISs entered the integration phase [10], focusing on network interconnection. The NRCMS information systems at the county level in 24 Chinese provinces were integrated into MIISs, while the situation remained unchanged in the remaining seven Chinese provinces. On the one hand, China continued to expand the coverage of the insured groups, and MIISs continued to expand in coverage; the systems further emphasized the interconnection among the central government, provinces, cities, and counties. As well, data were centralized from the bottom to the top, and data applications were further deepened [11,12]. Improvements included the following: the in-depth application of intelligent audit was realized; the diagnosis, treatment, and service behaviors of medical institutions, physicians and doctors, and pharmacies and drugstores were put under monitoring [13]; settlement and reimbursement for cross-provincial hospitalization were extensively carried out; people participating in the medical insurance scheme could transfer their medical insurance coverage to other provinces; public services, such as payment and direct reimbursement and settlement, became more convenient [14]; and there were more and more big data–based analyses and utilizations, as well as applications and studies on macro decision making [15].

In Phase III, information systems were built in line with the functions of the new NHSA, including the new medical security bureaus at all levels. In the context of big data—in addition to the realization of such functions as management and handling of medical insurance—price administration of medical services, and bidding and procurement of drugs, more data analysis and utilization can be realized; this would provide support for actuarial service for insurance and policy formulation. The new information systems will be built with 14 subfunctions in four categories. First, information systems in the category of internal management include the internal unified portal system and the process control system. Second, information systems in the category of business management include the basic information management system, the credit rating management system, the medical and drug price management system, and the payment method management system. Third, information systems in the category of production handling include the system for basic management of business handling and public services, the bidding and procurement system for drugs and medical supplies, and the cross-provincial transregional medical service management system. Fourth, information systems in the category of data analysis include the operation monitoring system, the intelligent regulation system, and the macro decision-making application system based on big data mining technology.

### Objective

This paper aims to collect and analyze the medical insurance informatization policies throughout the years, survey the status quo of provincial MIIS-building in China by means of questionnaires about the building of MIISs and their relevant policies, provide references and suggestions for the top-level design and implementation of the information systems in the transitional period of China’s MIS reform, and provide a reference for the building of MIISs in developing countries.

## Methods

### Overview

**Figure 2:**
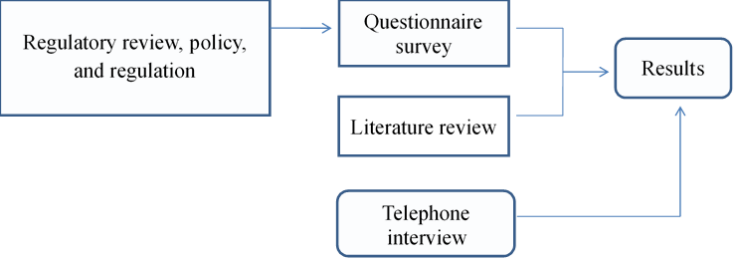
Flowchart of methods used in the study.

We conducted a literature review to learn about the relevant policies, regulations, methods, and application results of MIIS-building practices at home and abroad. We conducted a policy analysis by collecting the laws, regulations, and policy documents on medical insurance and medical insurance informatization issued from 1998 to 2020. We conducted a questionnaire survey by designing and distributing questionnaires to the medical security bureaus of 31 Chinese provinces. We conducted telephone interviews to verify the results of the questionnaires and to understand the focus issues concerning the building of China’s national MIISs during the period of integration and transition of China's MIS. Figure 2 shows the design of the methods used for this paper for the policy analysis, literature review, questionnaire survey, and telephone interview.

### Literature Review

We used the following keywords to search in literature databases, including PubMed and ScienceDirect: “medical insurance information system,” “health insurance information system,” “claim data,” “health information system,” “HIPAA” (Health Insurance Portability and Accountability Act), “privacy policy,” “big data,” “health information exchange,” “health information standard,” and “health insurance database.” We also used the following keywords to search in the China National Knowledge Infrastructure platform, a Chinese literature database: “basic medical insurance information system,” “new rural cooperative medical scheme information system,” “medical insurance for urban employees information system,” “medical insurance information system,” “medical insurance database,” “big data,” and “medical insurance.” The publication dates of literature searched ranged from January 1998 to September 2019.

### Policy Review

#### Policy List

We searched the contents of the policies and technical standards related to China’s medical insurance from the policy and regulation columns on the official websites of the Chinese Government, the NHSA, the National Health Commission of the PRC, the Ministry of Human Resources and Social Security of the PRC, the NDRC of the PRC, and the Ministry of Civil Affairs of the PRC, among others; from these, we collected 124 content entries in total. In addition, we also searched the documents of foreign policies related to medical insurance from the official websites of the US Centers for Medicare & Medicaid Services, the US Department of Health and Human Services, and the French Ministry of Social Affairs and Health, among others; from these, we collected more than 30 content entries in total. Table 1 shows the list of 14 entries of representative policies and regulations [16-32] closely related to medical insurance informatization among the 124 entries of policy documents from China.

**Table 1:** List of China’s policies and regulations related to medical insurance informatization from 1998 to the present.

Regulation and policy names		Year (document No.)	Summary of informatization document
**Decision on Establishing the Basic Medical Insurance System for Urban Employees^a^** [16]			
	1. Key points of building the planning for the labor and social insurance management information system [17]	1998 (LSBH[1998]138)	To standardize the labor and social insurance management information system, of which the contents under management involve the microinformation of laborers, enterprises, and other labor organizations; strengthen the management of personnel, wages, job positions, labor relations, and social insurance relations; and solve fundraising, payment, and other handling of businesses
	2. Notice of guidance on the building of the basic medical insurance management information system for urban employees [18]	2000 (LSTH[2000]30)	To establish network connections with designated medical institutions, designated retail pharmacies, banks, tax departments, and other relevant departments through establishing a computer management information system; focus on the unification of classification standards, interface standards, and network transmission standards; and promote the application of identity recognition media for ID cards
	3. Opinions on comprehensive implementation of the Jinbao Program for unified building of a labor security information system [19]	2003 (LSBH[2003]174)	To plan to establish a labor security information system with unified standards, which covers business handling of medical insurance, unemployment insurance, and industrial injury insurance; emphasize data centralization at the provincial and national levels; and build data centers at different levels
**Opinions of the Ministry of Health, Ministry of Finance, and Ministry of Agriculture on the Establishment of a New Rural Cooperative Medical Insurance System^b^** [20]			
	4. Basic specifications of the information system for the New Rural Cooperative Medical System (NRCMS) (trial) [21]	2005 (WBNWF[2005]108)	To build an information system for handling the business of the NRCMS at county level, which comprises six modules, including NRCMS participation management, medical service compensation management, fund collection and expenditure management, accounting, and statistical analysis
**Guidelines on Launching a Pilot Program of Basic Medical Insurance for Urban Residents^c^** [22]			
	5. Notice on carrying out unified implementation of some application software for the Jinbao Program [23]	2008 (RSTH[2008]284)	To build private networks at national, provincial, and municipal levels; upgrade the stand-alone version to a networking operation; and unify the core software functions of the financial exchange library software, fund statement software, fund regulation software, and transregional national service hotline software
	6. Basic specifications of the management information system for the NRCMS (2008 revised edition) [24]	2008 (WBNWF[2008]127)	To upgrade the *Basic specifications of the information system for the NRCMS (trial)* and to put forward the basic architecture for building the information system for the NRCMS China-wide, the building specification of physical environment and infrastructure, the functional specifications of the information system, and the datasets and code specifications for cross-system data exchange, among others
	7. Notice on carrying out unified upgrading implementation of some application software for the Jinbao Program [25]	2008 (RSXXH[2008]2)	To revise and upgrade the *Notice on carrying out unified implementation of some application software for the Jinbao Program*, improve the building of a network based on nationwide connectivity, and accelerate data centralization at the national level
	8. The scheme for connectivity technology of the national-level information platform for the NRCMS (trial) [26]	2013 (WBNWH[2013]456)	To establish a national-level information platform for the NRCMS, establish a data exchange network connecting all provincial platforms, collect data from all provinces, and explore the functions of cross-provincial cost verification and reimbursement
	9. Notice of the General Office of the Ministry of Human Resources and Social Security on comprehensively promoting the intelligent monitoring of medical services for basic medical insurance [27]	2015 (RSTF[2015]56)	To carry out all-around intelligent monitoring of outpatient service, hospitalization, and drug purchase in pharmacies and drugstores through an information system, identify suspected violations, and then verify and handle such violations
	10. Notice of the General Office of the Ministry of Human Resources and Social Security on carrying out the building of the information system for registration of universal participation in insurance [28]	2015 (RSTF[2015]86)	To plan to further raise the level of information system building at the provincial level, promote social security cards, advance the building of the information system for registration of universal participation in insurance, and push data at the national level; in addition, establish a list of qualifications for developers undertaking the building of information systems for the medical security industry
	11. Implementation plan for networked settlement and reimbursement for transregional hospitalization in the national NRCMS [29]	2016 (GWJCF[2016]23)	To build a national NRCMS network for cross-provincial hospitalization settlement and realize the functions of hospital visits, referral, hospitalization registration, and discharge reimbursement for patients participating in the NRCMS
	12. Notice of the General Office of the Ministry of Human Resources and Social Security on accelerating the building of a cross-provincial hospitalization settlement system [30]	2016 (RSTF[2016]185)	To realize functions such as filing of off-site urban employees participating in medical insurance and settlement for discharge reimbursement
**China established the National Healthcare Security Administration (NHSA)^d^** [31]			
	13. Notice of the NHSA on printing and distributing the guidance on medical security informatization [32]	2019 (YBF[2019]39)	After its inception, the new NHSA proposed to establish a new, nationally integrated, standard-unified Medical Security Information System, which covers such functions as management of basic knowledge (eg, dictionary directory), business handling management, public service management, and data analysis management; the system developed 15 content standards, such as diagnosis and surgery standards, among others
	14. Notice of the NHSA on carrying out the pilot work of medical security informatization	2019 (YBF[2019]22)	The NHSA selected 16 pilot provinces for implementing information system functions first, carrying out 15 standards, and realizing cross-provincial business linkage and public services through the building of the national platform

^a^In 1998, the State Council of the People's Republic of China (PRC) issued the Decision on Establishing the Basic Medical Insurance System for Urban Employees (No. GF[1998]44), enforcing a basic medical insurance system (MIS) for urban employees throughout China, and exploring the establishment of socialized medical insurance for the population with labor and employment relationships [16].

^b^In 2003, the State Council of the PRC issued the Opinions of the Ministry of Health, Ministry of Finance, and Ministry of Agriculture on the Establishment of a New Rural Cooperative Medical Insurance System (No. ZF[2002]13) [20], aiming to establish an MIS for rural residents on a pilot basis.

^c^In 2007, the State Council of the PRC issued the Guidelines on Launching a Pilot Program of Basic Medical Insurance for Urban Residents [22], aiming to establish a basic MIS for nonemployed urban residents and those with nonfixed-employment relationships.

^d^In 2018, China reformed its state institutions and established the NHSA, which functions to unify the administration of the basic MIS [31].

#### Policy Analysis

We analyzed the evolution of policies from the five main elements that constitute management information systems (ie, organizational structure, process, data, business rules, and system functions).

### Questionnaire Survey

We surveyed the health care security bureaus, or their information centers, of 31 Chinese provinces in terms of network infrastructure, identity recognition media, information system functions, data centers, standards, and specifications. We sent out 31 questionnaires and recovered all 31 of them.

### Telephone Interview

We interviewed more than 30 persons by telephone from the departments of health care security administration, medical insurance handling management, health administration, and hospital management, among others. Issues discussed during telephone interviews included the functions urgently needed by MIISs and concerns about information system building in the integration period of MISs.

## Results

### Analysis of Functions Realized by Historical Information Systems

#### Business Modeling for China’s MIISs

**Figure 3:**
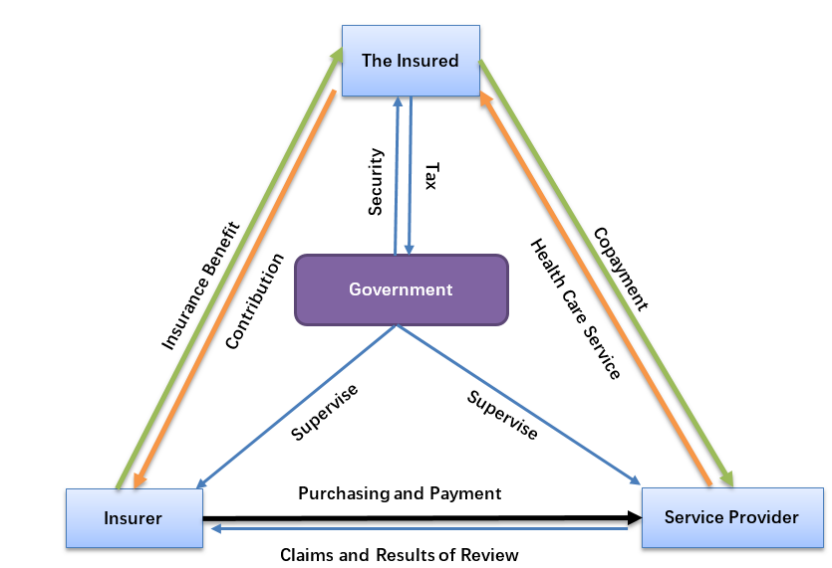
Business model chart of China’s medical insurance information systems (MIISs), modified from the the National Health Information Database of the National Health Insurance Service in South Korea (Cheol Seong et al, 2017).

China’s MIISs support the handling and management of the medical insurance business. The operation of the Chinese MIS is led by the government. Funds can be raised through the taxation of people with fixed employment, such as urban employees. People who have no fixed employment, such as farmers and urban residents, pay cash directly to medical insurance handling institutions. Medical service providers provide medical services to patients, handling institutions provide medical institutions with the settlement of medical insurance funds, and handling institutions manage patient reimbursement and other services. Figure 3 shows the business model, the main elements of China’s MIISs, and the interrelationship therein [4]. Medical insurance administrative departments, medical insurance handling institutions, medical service providers, and insured persons play different roles and interact with each other. Medical insurance administrative departments make and regulate medical insurance policies, while medical insurance handling institutions, as the specific implementers of medical insurance policies, provide medical insurance handling and management services for the insured and medical service providers. Affected by the functional adjustment of the national-level administrative department, the administrative level and management authority of China’s medical insurance administrative departments as well as medical insurance handling institutions have changed several times, but there has been no significant change in the substance of their respective functions.

#### Analysis of the Evolution of Information System–Related Policies

##### Organizational Structure Analysis

Organizational structure includes medical insurance administrative departments, medical insurance handling institutions, medical service providers, and patients, among others. The policy under document No. LSBH[1998]138 shows that social security is administered by two departments—the Ministry of Personnel of the PRC and the Ministry of Labor and Social Security of the PRC—covering enterprises and employees with employment relationships; medical services are provided mainly by medical institutions as well as pharmacies and drugstores subject to designated administration. The policies under document Nos. ZF[2002]13 and GF[2007]20 include rural and urban residents without employment relationships in the coverage for basic medical insurance. The policy under document No. RSTH[2008]284 shows the merger of the Ministry of Personnel of the PRC, the Ministry of Labor of the PRC, and the social security bureaus of China into one ministry, namely, the Ministry of Human Resources and Social Security of the PRC, which is in charge of medical insurance for urban employees and urban residents. The policy under document No. RSTF[2015]56 emphasizes universal coverage of the MIS, aiming for covering all those who should be covered. The policy under document No. EBF[2019]39 shows that the administrative department for basic medical insurance was merged into the NHSA of the PRC, which covers the administration of drug suppliers and manufacturers, who need to participate in bidding for drug procurement.

##### Business Process Analysis

Since the issuance of the policy under document No. RSXXH[2008]2, as shown in the business model chart of China’s MIISs (see Figure 3), the medical insurance business process focuses on payment by insured persons as well as hospitalization and reimbursement at the place of insurance participation. Medical institutions provide medical services and settle accounts with medical insurance handling institutions. Medical insurance handling institutions are mainly responsible for raising funds and clearing up of medical expenses with medical institutions as well as pharmacies and drugstores. The Chinese government formulates policies and supervises all stakeholders. Since the issuance of the policy under document No. RSXXH[2008]2, the Chinese government began to pay attention to transregional transfer and continuation of insurance participation relationships as well as hospitalization and reimbursement; especially since the issuance of the 2016 policy, China has launched a large-scale promotion of cross-provincial hospitalization reimbursement, in order to meet the needs of population mobility and employment all over the country. Since 2018, drug procurement through bidding has been included in the scope of medical insurance administration.

##### Data Analysis

Analysis by data type covers administrative divisions, enterprise entities, hospitals, pharmacies and drugstores, fundraising, hospitalization behaviors, fund reimbursement, and directory data, such as disease diagnosis, drugs, and medical devices. Analysis by data level shows the following: before 2010, data flowed, were stored, and were utilized mainly at and below the prefecture level; since the issuance of the 2018 policies under document Nos. RSXXH[2008]2 and WBNWF[2008]127, data gradually flow toward the provincial and national levels; the 2016 policies under document Nos. GWJCF[2016]23 and RSTF[2016]185 aim to realize cross-provincial data flow through a national-level platform and support collaborative businesses, such as transregional transfer and continuation of insurance participation relationships and hospitalization reimbursement.

##### Business Rules Analysis

Business rules have adapted to the medical insurance administration functions and covered the insurance participation and hospitalization reimbursement for urban employees in 1998, for rural residents in 2003, and for urban residents in 2007. Meanwhile, the policies of insurance participation and medical insurance reimbursement were embedded into information systems, in order to standardize the behaviors of medical insurance participation, medical insurance handling, and hospitalization reimbursement. The policy under document No. RSTF[2015]56 emphasizes the use of big data analysis technology to carry out intelligent monitoring of patients’ hospitalization behaviors, doctors’ diagnosis and treatment behaviors, and handling institutions’ handling behaviors. The policy under document No. YBF[2019]22 incorporates drug procurement bidding rules into information system administration; incorporates such payment methods as diagnosis-related groups into the process of patient hospitalization, reimbursement, and fund clearing, step by step; carries out extensive interconnection with such departments as health, taxation, and public security, as well as with such entities as banks and insurance companies; and gradually establishes a nationwide medical insurance credit system.

##### Analysis of Information System Functions

Before the issuance of the 2018 policies under document Nos. RSXXH[2008]2 and WBNWF[2008]127, China’s MIISs were mainly responsible for managing basic functions for patients, medical institutions, and medical insurance handling institutions in terms of insurance participation, payment, reimbursement, and fund clearing, as well as managing such standards as disease diagnosis standards and drug lists. Since the issuance of the 2015 policy, China’s MIISs gradually strengthened the collection and utilization of data and realized business supervision. The policy under document No. YBF[2019]39 shows that big data gradually plays a role in promoting the fine management of medical insurance and providing evidence-based support for formulation and evaluation of policies.

#### Main Functions of China’s MIISs

**Figure 4:**
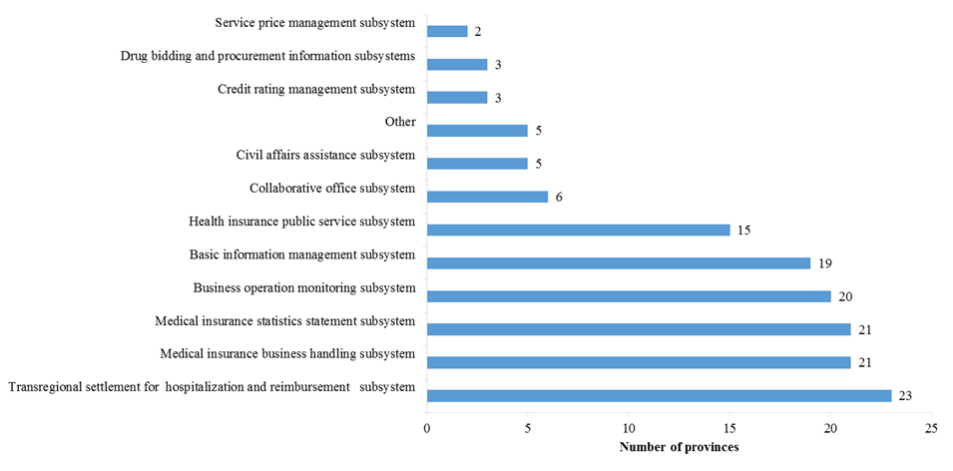
Distribution of business function modules used in various provinces in China according to questionnaire feedback.

The existing MIISs mainly function to describe the status quo before the institutional integration (ie, before 2018). The related functions of the MIISs are scattered throughout multiple ministries and commissions. The information systems related to these functions include the medical insurance handling subsystem, the civil affairs assistance subsystem, and the drug bidding and procurement subsystem, among others. Figure 4 shows the distribution of business function modules used in various provinces in China according to questionnaire feedback. Details of each module are shown below:

1. The medical insurance statistics statement subsystem can be used to understand the data regarding insured persons, insured entities, insured rate, fund collection and expenditure, number of hospital visits, average reimbursement amount, and compensation ratio in each region.
2. The medical insurance business handling subsystem functions to manage the insured entities, insured persons, employment relationships, payments by entities and individuals, medical insurance card issuance, personal identity recognition, hospital visits by insured persons, handling of reimbursement and compensation procedures, and compensation for serious illness insurance—if the individual out-of-pocket payment exceeds a certain amount, the serious illness insurance will compensate them again—among others.
3. The civil affairs assistance subsystem functions to manage the groups who receive civil affairs assistance, the distribution of medical assistance funds, and the compensation for medical services—compensation will be provided again on the basis of the basic medical insurance compensation—among others.
4. The drug bidding and procurement information subsystems are established by the responsible administrative departments: development and reform commission, health commission, health care security bureau, etc. The responsible provincial administrative departments, on behalf of hospitals, negotiate with drug manufacturers and suppliers and complete the tender process through this information system. Some provincial administrative departments directly pay funds to drug manufacturers and suppliers, while funds for drug supply are paid by hospitals to drug manufacturers and suppliers in some other provinces.
5. The transregional hospitalization and settlement subsystem supports transregional or transprovincial reimbursement of insured persons and covers the following functions: hierarchical referral, identify recognition and identification of insured status, discharge settlement, and window-based reimbursement, as well as fund clearing among transprovincial handling institutions.
6. The service price management subsystem functions to monitor the sales prices of medical institutions and pharmacies and drugstores, as well as to analyze their changing trends, so as to provide a reference basis for price formulation and payment standards of medical services, drugs, and medical devices.
7. The credit rating management subsystem functions to manage credit rating for the insured entities, insured persons, medical institutions, pharmaceutical production and circulation enterprises, and medical workers, and to establish a credit management system for medical services and medical insurance handling.
8. The public service subsystem for medical insurance caters to insured persons; provides self-services, such as insurance participation, payment, and referral filing; supports mobile online payment; and allows the inquiry of personal historical behaviors, such as insurance participation and hospital visits, so as to assist health management.
9. The basic information management subsystem functions to manage the qualification information for designated medical institutions and designated pharmacies and drugstores so they may join the MIS. It also functions to manage the information of medical workers and maintain the dictionary codes for disease diagnosis, diagnosis and treatment services, drugs, and consumables.
10. The business operation monitoring subsystem functions to monitor the compliance of medical services and the balance of revenue and expenditure of medical insurance funds, among others, by collecting data on insurance participation, medical treatment, treatment behavior, reimbursement and compensation, among others. It also functions to make forecasts on partial trends of medical insurance participation, fund collection, and expenditure.
11. The collaborative office subsystem integrates all office automation work within a jurisdiction into the information system for unified management, such as routine management of mail delivery as well as drafting, approval, and receipt of official documents, among others.

The 11 business function modules above, just like components of a jigsaw puzzle, build up the framework of MIISs in each province. Some functions, such as electronic medical record management, budget management, financial management, and account book management, have also been mentioned in some regions.

### Analysis of Infrastructure and Identity Recognition Media

#### Networking Mode

**Figure 5:**
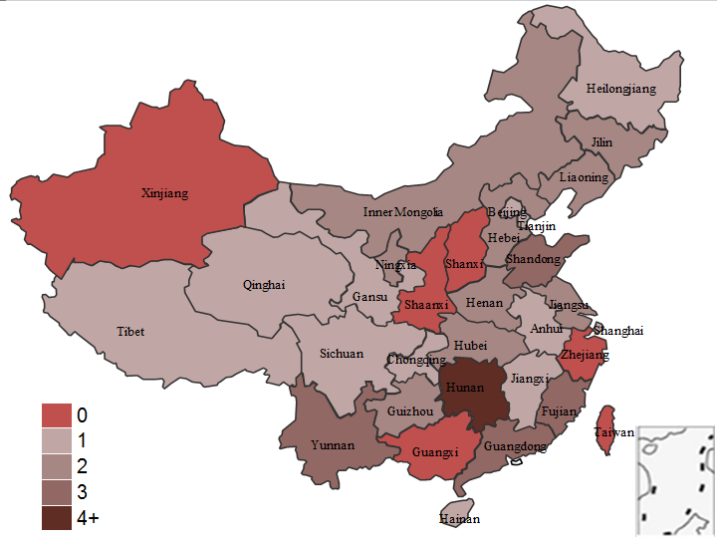
Analysis of networking modes adopted by medical insurance information systems (MIISs) in each province in China.

Four main networking modes include the following: fiber optic private network (FOPN), e-government extranet (EGE), virtual private network (VPN), and the internet. FOPN features high-security performance, good transmission performance, and high cost. EGE connects medical insurance handling institutions at all levels and features lower cost and better security performance, but it is only connected to government agencies and is not yet connected to hospitals. VPN establishes a virtual safe channel based on the internet and features low cost and better security performance. The internet is characterized by good transmission performance and low cost, but its security performance is low. Figure 5 shows the distribution of the four networking modes above.

A total of 26 provinces of China, including provinces, autonomous regions, and municipalities directly under the central government, have responded with their networking modes; only Hunan Province has used all four modes. Four provinces—Fujian, Shandong, Guangdong, and Yunnan—use three networking modes. Nine provinces at all levels—Hebei, Inner Mongolia, Liaoning, Jilin, Jiangsu, Henan, Hubei, Guizhou, and Ningxia—use two networking modes. A total of 12 provinces—Beijing, Tianjin, Heilongjiang, Shanghai, Anhui, Jiangxi, Hainan, Chongqing, Sichuan, Tibet, Gansu, and Qinghai—use one networking mode.

Regarding the main networking modes used, 23 out of 31 (74%) provinces use FOPN; Anhui, Tibet, and Hainan are the only provinces that do not use FOPN for networking. Six provinces—Fujian, Henan, Hunan, Guangdong, Yunnan, and Ningxia—also use EGE for networking.

#### Identity Recognition Media

There are mainly two kinds of identity recognition media: ID cards and social security cards. A total of 20 provinces out of 31 (65%) support both ID cards and social security cards for identity recognition, while some provinces, such as Inner Mongolia and Chongqing, support only one kind of media for identity recognition.

### Analysis of Data Storage and Data Utilization

#### Interconnectivity of Information Systems

**Figure 6:**
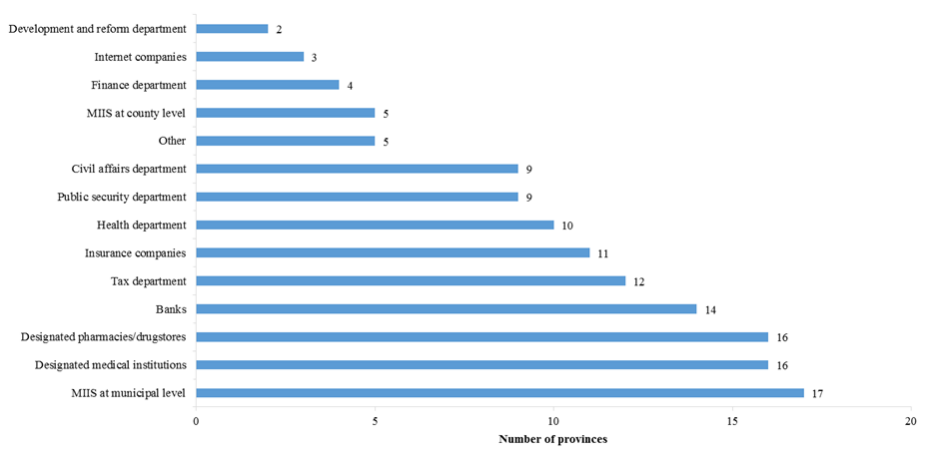
The interconnection between the medical insurance information systems (MIISs) and the surrounding information systems in each province.

A provincial MIIS vertically connects municipal MIISs and regional pharmacy information systems; it exchanges referral and transregional hospitalization settlement data with municipal MIISs and exchanges drug purchase information via personal accounts with designated pharmacies and drugstores. A provincial MIIS horizontally connects the information systems of such departments and entities as tax, finance, development and reform, health, civil affairs, public security, banks, insurance companies, and internet companies. This MIIS exchanges information with different departments and entities as follows: (1) it exchanges information on collection and payment of insured expenses with the tax department, (2) it exchanges electronic medical record information with the health department, (3) it exchanges personal identity and credit information with the public security department, (4) it exchanges medical insurance fund transfer information with banks, (5) it exchanges poverty alleviation and social assistance information with the civil affairs department, (6) it exchanges serious illness insurance information with insurance companies, and (7) it exchanges online payment information with internet companies, among others. MIISs in some provinces also exchange information with departments of audit, industry, and commerce, among others. Figure 6 shows the connection between provincial MIISs and peripheral information systems.

In 55% (17/31) of the regions in China, MIISs at provincial and municipal levels were networked and have gathered basic medical insurance data. Provincial MIISs in 11 provinces out of 31 (35%) exchange data with insurance companies; the main data item exchanged includes the secondary compensation information for serious illness insurance following basic medical insurance compensation. Provincial MIISs in 12 provinces out of 31 (39%) exchange data with tax departments. In adaption to the adjustment to their functions in the medical insurance reform, the tax departments are responsible for the collection and payment of insurance premiums.

#### Data Storage Types at Provincial Information Centers

Feedback from 24 provinces in China shows that some types of medical insurance data are stored in these provinces by establishing provincial-level medical insurance data centers; such data centers are under construction in two provinces: Shandong and Henan. Seven provinces did not fill out the questionnaire. Many data storage types included insurance participation, payment, settlement, and expenses. Among these types, information about insured individuals and insured entities accounted for 92% (22/24), the highest share; information related to payment, settlement compensation, details of outpatient and inpatient expenses, and cross-provincial hospitalization accounted for over 83% (20/24), the second-highest share. No provincial-level medical insurance data center has stored the procurement, sales, and inventory data from designated pharmacies and drugstores. The storage is also very limited for procurement, sales, and inventory data from hospitals (1/24, 4%) and data from bidding and procurement of drugs (3/24, 13%). Efforts should be made to further expand the scope of data exchange and sharing, so as to match the functions of MIISs in terms of bidding and procurement of drugs as well as use of drugs and consumables. Feedback from all provinces that were surveyed shows that Heilongjiang Province has stored the highest number of data types (15/17, 88%); this province has covered all data types listed in Multimedia Appendix 1 except the procurement, sales, and inventory data from hospitals as well as pharmacies and drugstores. A total of 76% or more of relevant information has been stored in such provinces and municipalities as Tianjin (13/17, 76%), Shanghai (13/17, 76%), Guangdong (14/17, 82%), Hainan (13/17, 76%), and Guizhou (13/17, 76%). According to the feedback from the questionnaires, the provincial-level medical insurance data center in Hubei Province has collected the payment information on medical insurance participation of urban and rural residents in the whole province, as well as information on medical insurance treatment for urban and rural residents in individual prefectures and cities, but it did not fill out the information about other types of data storage. Henan Province did not fill out information about other data storage types except for data related to insurance participation and payment. As a result, these two provinces—Hubei and Henan—have a relatively lower percentage of data storage types. In those provinces with high economic development levels, solid informatization foundations, and lower pooling regions, their provincial-level medical insurance data centers have stored relatively more types of data; however, as a whole, the degrees of concentricity and comprehensiveness of medical insurance data are still low. Multimedia Appendix 1 shows the corresponding matrix of provincial medical insurance data centers and data storage types.

### Analysis of Data Utilization as well as Security and Privacy Protection Policies

China’s medical insurance data are mainly used for business handling; however, these data have not been fully exploited. Through the Medicaid Statistical Information System database [33], the United States supports the formulation of Medicaid fundraising and compensation policies. France built its national database EGB (Echantillon Generaliste des Beneficiaires) [34], conducts research on the effects of drugs, and conducts research and development on new drugs through claims data [35]. South Korea built its National Health Insurance Research Database, which functions to make secondary compensation for catastrophic expenditures; it also provides specific datasets to researchers for scientific research, especially for public health governance and infectious disease monitoring through data correlation [36]. The medical insurance database of Taiwan is used for research on cancer treatment [37].

In terms of policies and regulations related to medical insurance information security and privacy protection, the HIPAA was passed by the US Congress in 1996; its applicability was adjusted continuously, especially in the era of big data [38]. In 2016, the European Union issued the General Data Protection Regulation, which stipulates in detail the collection, transmission, processing, and utilization of medical security or health information [39]. China has not yet issued a special law or regulation on security and privacy protection of health information or medical insurance information; some of the existing Chinese policies are scattered among the Social Insurance Law of the PRC, the Law of the PRC on Basic Healthcare and Health Promotion, the Law of the PRC on the Prevention and Treatment of Infectious Diseases, and the Provisions of the PRC on the Disclosure of Government Information. However, the length of such content is extremely limited; there are provisions of a framework nature, but there are no acts or laws with operability.

### Urgent Needs in Various Regions During the Period of Integration and Transition of MISs

The information system building model is guided by national top-level planning; the deployment level of information systems should be higher than the unified pooling level of medical insurance funds. In addition, attention should be paid to data coding and standardization and building efforts should be made on the basis of the original information system building, in order to save unnecessary investment.

In terms of functions of information systems, efforts should be made to strengthen the informatization support for the reform of payment mode, in order to adapt it to the promotion of prepayment modes such as diagnosis-related groups. In the era of big data, work should be done to establish an intelligent medical insurance audit system, which will function to monitor behaviors (eg, medical treatment and fund compensation) through algorithms, such as data machine learning, and will identify such events as medical insurance fraud and abuse. Meanwhile, work should also be done to establish a credit database containing information on lawbreakers to be able to take certain punishment measures against such persons.

In the context of the universal application of mobile phones, efforts should be made to provide more self-services for the public, such as online hospitalization appointment, mobile payment, self-service insurance participation and payment, policy access, and information inquiry.

## Discussion

### Building Model of China’s Basic MIISs in the Future

In the future, we should overcome the disadvantage of the lack of overall planning for the building of China’s MIISs in the first and second phases and arrange for the building of MIISs and hospital information systems at the national, provincial, and municipal levels on a unified basis. We should establish a deployment mode higher than the fund pooling level that is at least not lower than the municipal deployment level. In addition, we should try our best to realize provincial deployment and to deploy prefectural and municipal information systems by utilizing the cloud computing model. There are more than four networking modes, which need further planning to establish a safer and faster private network mode. In terms of identity recognition media, this should expand to identity recognition based on electronic ID cards; moreover, by adopting the QR (Quick Response) code, we can use mobile phones to recognize identities. Information system building standards should be formulated and issued first, covering the planning of medical insurance business, specification of information system modules, and standard codes for data exchange, among others.

### Information Exchange and Data Utilization

Information exchange involves the integration of internal information systems in the MIS, the collection of data at different levels, and the exchange of data within fields or between different fields. The MIS internal information systems receiving close attention exceed the 11 subsystems listed above in the *Main Functions of China’s MIISs* section. These systems are designed and developed by many developers; as a result, they have diversified data structures and codes. First, internal integration of these information systems shall avoid repeated investment. Second, we should carry out the mode of deployment of MIISs at the national, provincial, prefecture, and municipal levels; unify the standards for data exchange; and realize bottom-up collection and gathering of data. Third, we should realize data exchange with the information systems in other fields, clarify the operation specifications for business links, and realize business collaboration through data exchange; for instance, exchanging data with the tax department to confirm the qualification of patients to participate in insurance and medical insurance payment, and exchanging medical record data with the health department. Through such practice as standardization of health information exchange data [40] and Federal Enterprise Architecture Framework business [41], we will realize the business interoperation of different systems, such as fundraising and payment to the tax department, reimbursement for inpatients and outpatients at health departments, designated institution certification at industry and commerce departments, and claim settlement at commercial insurance companies.

### Balancing the Stake Between Informed Consent for Privacy Protection and Data Mining and Utilization in a Context of Big Data

MIISs cover a massive amount of heterogeneous data, which have the typical 4-V characteristics of big data: volume, variety, velocity, and veracity. For example, residents’ participation in medical insurance and hospital visits involve detailed identity information of individuals, health information, economic status, invoice images, and other relevant data. China needs to formulate special-purpose laws for the security and privacy protection of medical insurance information. China also needs to clarify the connotation of medical security information, the rights and interests of the public, the scope of security and privacy protection, the operation requirements for the right to be informed and information disclosure, the contents of exceptional protection of safe harbor, infringements, and punishments, among others, by referring to the HIPAA of the United States and the Personal Information Protection and Electronic Documents Act (PIPEDA) of Canada.

We should balance the stake between *security and privacy protection* and *data analysis and utilization*. New models for assisting in disease diagnosis and treatment have been identified from the big data of health records through utilization of artificial intelligence technology. These new models have been widely used in medical innovation, which involves patients’ health histories, treatment methods, and treatment results, and are even associated with such information as genetics; in particular, the association with multisource data makes privacy protection more difficult. We should ensure users’ rights of informed consent and enable patients to feel comfortable in providing data for scientific research without degrading safety protection measures. This represents a direction of collection and utilization of medical insurance information, rather than transitional privacy protection [42].

### Lessons From COVID-19 for Building of MIISs

China has recently developed hospital information systems. China’s MIISs have established a mechanism of data exchange and sharing with each hospital, in order to meet patients’ health needs and facilitate settlements and expense compensations. However, China’s regional health information platforms of health departments and authorities are relatively isolated, and a normalized mechanism of data exchange with hospitals has not yet been established. Although China has established the world’s most extensive surveillance system for infectious diseases, this system is mainly based on a bottom-up reporting approach by manual entry and form filling; as a result, China’s system fails to exchange data with hospital information systems in real time [43]. Reporting time is delayed; the system needs a process that begins with identifying a suspected case of an infectious disease and leads to case confirmation, thereof. The source of data generation has no authority to publish information, which has resulted in delays in reporting cases of coronavirus disease 2019 (COVID-19) after case identification as well as delays in information publication to the public after reporting to the central government. During the whole process, the early warning mechanism of the Infectious Diseases Information Network failed to work effectively, which resulted in serious decreases in disease prevention and early warning. This suggests the following: China’s new MIISs should be closely combined with its medical and health information systems; efforts should be made to exert the roles of numerous medical institutions as parts of a network foundation, in order to gather data from hospitals to be transferred to the national-level data center in a timely manner; and work should be done to establish a computer-based early warning model, in order to detect the sudden states and development trends of infectious diseases and public health events in various regions [44]. Through information connection between MIISs and health systems, we will be able to capture information about patients’ hospitalizations at medical institutions in a timelier manner and more efficiently. Then, through dynamic analysis and summarized reports of data regarding disease types and expenses, etc, we will be able to identify risk factors in a prospective manner, so as to maintain the safety of medical insurance funds.

### Conclusions

China’s MIISs are the most extensive information systems that could allow network foundations to connect medical institutions. Over the past 20 years, after the three phases of development, China’s MIISs have played an important role in medical insurance business management and reimbursement, and have provided strong support for the operation of the world’s largest medical security system. Particularly in terms of settlements for transregional hospitalization and reimbursements, China’s MIISs have enabled extensive data exchange among the central government, provinces, prefectures, municipalities, cities, and medical institutions, and have realized transprovincial business collaboration. In many developing countries, information system building is an indispensable element to realize universal health coverage and to continuously improve their respective medical security systems. The analysis on the functions, advantages, and disadvantages of China’s MIISs at different phases has a sound significance of reference. Currently, China’s MIISs are in a period of transformation and transition. In terms of the top-level design and planning of China’s national medical insurance informatization, as well as the redeployment and reimplementation of information systems, it is necessary to further consider such focal issues as normalization of business, standardization of data, and interoperation of information systems. In 2019, the outbreak of COVID-19 revealed a poor interoperability between the MIISs and the health information systems. Due to privacy protection and other reasons, data sharing with the public health information network was insufficient, and big data technology was not fully utilized to analyze medical insurance data and provide early warning services for public health. In the future, more detailed laws, regulations, and policies should clearly set forth the contents and ways of exchanging and sharing medical insurance data. The implementation of security and privacy protection policies of MIISs will further improve the degree of trust from individuals, medical service providers, and public health institutions in the information systems.

## Appendix

Multimedia Appendix 1 List of data storage types used by the medical insurance data centers from each province in China.

## Abbreviations

COVID-19: coronavirus disease 2019
EGB: Echantillon Generaliste des Beneficiaires
EGE: e-government extranet
FOPN: fiber optic private network
HIPAA: Health Insurance Portability and Accountability Act
MIIS: medical insurance information system
MIS: medical insurance system
NDRC: National Development and Reform Commission
NHSA: National Healthcare Security Administration
NRCMS: New Rural Cooperative Medical System
PIPEDA: Personal Information Protection and Electronic Documents Act
PRC: People's Republic of China
QR: Quick Response
VPN: virtual private network
