# Supplementary material for: Medical Insurance Information Systems in China: Mixed Methods Study
Source: JMIR Med Inform. 2020 Sep 1;8(9):e18780. doi: 10.2196/18780 (PMC7492979; doi:10.2196/18780)
Supplement: Multimedia Appendix 1 [file medinform_v8i9e18780_app1.docx]

Multimedia Appendix 1. List of data storage types used by the medical insurance data centers from each province in China.

| Data storage type | Province^a,b^ (n=24) | | | | | | | | | | | | | | | | | | | | | | | | n (%) |
| --- | --- | --- | --- | --- | --- | --- | --- | --- | --- | --- | --- | --- | --- | --- | --- | --- | --- | --- | --- | --- | --- | --- | --- | --- | --- |
|  | B | T | H | IM | L | J | He | S | A | F | Ji | Sh | Hen | Hub | Hun | G | Ha | C | Si | Gu | Y | Ga | Q | N |  |
|  | | | | | | | | | | | | | | | | | | | | | | | | | |
| Basic information on insured entities and persons in the whole province | ✓ | ✓ | ✓ | ✓ | ✓ | ✓ | ✓ | ✓ | ✓ | ✓ | ✓ | ✓ | ✓ | — | ✓ | ✓ | X | ✓ | ✓ | ✓ | ✓ | ✓ | ✓ | ✓ | 22 (92) |
| Information on fundraising of medical insurance funds in the whole province | ✓ | ✓ | ✓ | X | X | ✓ | ✓ | ✓ | ✓ | ✓ | ✓ | ✓ | ✓ | — | ✓ | ✓ | ✓ | ✓ | ✓ | ✓ | ✓ | ✓ | ✓ | ✓ | 21 (88) |
| Information on compensation paid by medical insurance funds to hospitals and insured persons | ✓ | ✓ | ✓ | ✓ | X | ✓ | ✓ | ✓ | ✓ | ✓ | ✓ | ✓ | — | — | ✓ | ✓ | ✓ | ✓ | ✓ | ✓ | X | ✓ | ✓ | ✓ | 20 (83) |
| Information on outpatient summaries of insured persons in the whole province | ✓ | ✓ | X | ✓ | X | ✓ | ✓ | ✓ | X | ✓ | — | — | — | — | X | ✓ | ✓ | — | ✓ | ✓ | ✓ | ✓ | ✓ | ✓ | 15 (63) |
| Information on first pages of inpatient medical records of insured persons in the whole province | ✓ | X | X | X | X | — | ✓ | X | X | ✓ | — | ✓ | — | — | X | ✓ | ✓ | — | X | ✓ | X | ✓ | ✓ | X | 9 (38) |
| Information on outpatient details of insured persons in the whole province | ✓ | ✓ | ✓ | ✓ | X | ✓ | ✓ | ✓ | ✓ | ✓ | ✓ | ✓ | — | — | X | ✓ | ✓ | ✓ | ✓ | ✓ | ✓ | ✓ | ✓ | ✓ | 20 (83) |
| Detailed information on hospitalization expenses of insured persons in the province | ✓ | ✓ | ✓ | ✓ | X | ✓ | ✓ | ✓ | ✓ | ✓ | ✓ | ✓ | — | — | ✓ | ✓ | ✓ | ✓ | ✓ | ✓ | X | ✓ | ✓ | ✓ | 20 (83) |
| Information on outpatient compensation for insured persons in the whole province | ✓ | ✓ | ✓ | ✓ | X | ✓ | ✓ | ✓ | ✓ | ✓ | ✓ | ✓ | — | — | X | ✓ | ✓ | — | ✓ | ✓ | X | ✓ | ✓ | ✓ | 18 (75) |
| Information on hospitalization compensation for insured persons in the whole province | ✓ | ✓ | ✓ | ✓ | ✓ | ✓ | ✓ | ✓ | ✓ | ✓ | ✓ | — | — | — | ✓ | ✓ | ✓ | — | X | ✓ | X | ✓ | ✓ | ✓ | 18 (75) |
| Information on civil affairs assistance for insured persons in the whole province | X | ✓ | ✓ | X | X | — | ✓ | ✓ | X | ✓ | — | — | — | — | X | ✓ | ✓ | ✓ | X | ✓ | X | ✓ | ✓ | X | 11 (46) |
| Information on drug bidding and procurement in the whole province | X | X | X | ✓ | X | — | ✓ | ✓ | X | X | — | — | — | — | X | — | X | — | X | X | X | — | X | X | 3 (13) |
| Information on designated medical institutions, medical workers, and agreements | ✓ | ✓ | ✓ | X | X | ✓ | ✓ | ✓ | X | X | — | ✓ | — | — | ✓ | ✓ | ✓ | — | ✓ | ✓ | ✓ | — | X | ✓ | 14 (58) |
| Hospitals’ purchase, sales, and inventory information | X | X | X | X | X | — | — | X | X | X | — | — | — | — | X | — | ✓ | — | X | X | X | — | X | X | 1  (4) |
| Information on designated pharmacies and drugstores and agreements | ✓ | ✓ | ✓ | X | X | ✓ | ✓ | ✓ | X | X | — | — | — | — | ✓ | ✓ | X | — | ✓ | X | ✓ | — | X | ✓ | 11 (46) |
| Pharmacies’ and drugstores’ purchase, sales, and inventory information | X | X | X | X | X | — | — | X | X | X | — | — | — | — | X | — | X | — | X | X | X | — | X | X | 0  (0) |
| Information on medical insurance poverty alleviation | X | ✓ | ✓ | X | X | — | ✓ | X | ✓ | X | — | — | — | — | X | ✓ | ✓ | ✓ | ✓ | ✓ | ✓ | ✓ | X | X | 11 (46) |
| Information about cross-provincial hospitalization | ✓ | ✓ | ✓ | X | ✓ | ✓ | ✓ | ✓ | ✓ | ✓ | — | ✓ | — | — | ✓ | ✓ | ✓ | ✓ | ✓ | ✓ | ✓ | ✓ | ✓ | ✓ | 20 (83) |
| Other | X | X | X | X | X | — | — | X | X | X | — | — | — | ✓ | X | — | X | — | X | X | X | — | X | X | 1  (4) |
| Number of data storage types (n=17), n (%) | 12 (71) | 13 (76) | 12 (71) | 8 (47) | 3 (18) | 11 (65) | 15 (88) | 13 (76) | 9 (53) | 11 (65) | 7 (41) | 9 (53) | 2 (12) | 1  (6) | 8 (47) | 14 (82) | 13 (76) | 8 (47) | 11 (65) | 13 (76) | 8 (47) | 12 (71) | 11 (65) | 11 (65) |  |

^a^B: Beijing; T: Tianjin; H: Hebei; IM: Inner Mongolia; L: Liaoning; J: Jilin; He: Heilongjiang; S: Shanghai; A: Anhui; F: Fujian; Ji: Jiangxi; Sh: Shandong; He: Henan; Hub: Hubei; Hun: Hunan; G: Guangdong; Ha: Hainan; C: Chongqing; Si: Sichuan; Gu; Guizhou; Y: Yunnan; Ga: Gansu; Q: Qinghai; N: Ningxia.

^b^Responses were Yes (✓), No (X), or no response (—).
